# Practical Gleanings

**Published:** 1856-04

**Authors:** Jno. R. Allen

**Affiliations:** Professor of Obstetrics, &c., College of Physicians and Surgeons, Iowa University


### IOWA MEDICAL JOURNAL.

VOL. III. KEOKUK, IOWA, MARCH AND APRIL, 1856. NO. 2.

### ORIGINAL COMMUNICATIONS.

### PRACTICAL GLEANINGS FROM BOOKS AND OBSERVATIONS.

BY JNO. R. ALLEN, M. D.,

Professor of Obstetrics, &c., College of Physicians and Surgeons, Iowa University.

Diseases of the Respiratory Passages.—These diseases are greatly multiplied, frequent, and often very grave; hence merit all the attention that can be given them, and may well be placed at the head of these gleanings.

Diseases of the Nasal Passages.—These are double; are the seat of olfaction and constitute an important part of the respiratory apparatus. They are thus interesting, in two ways, to the physiologist; but to the pathologist most, as a portion of the respiratory passages. The impairment of smell is to him but a symptom of a more important lesion, and is only thus submitted to his observation, and their importance is thus lost in the seriousness of the malady, of which they present but a symptom.

As a part of the breathing organs, they offer for his consideration, sometimes, important affections.

The following order, in describing the various diseases of these parts, will be adopted: 1. Epistaxis, a slight disease often, but sometimes very serious. 2. Simple Acute Coryza; or simple acute inflammation of the pituitary membrane. 3. Coryza Chro. Simple. 4. Ozena or Coryza Ulcerous.

EPISTAXIS, (NASAL HEMORRHAGE).—This is one of those hemorrhages which attaches to a great many persons, and to many pathological states. To study it, therefore, in all those cases in which it appears, would lead us beyond our intended limits. We are not now to consider it as an epiphenomenon of other diseases. We wish now to notice it as requiring its own proper interference of the art; a malady itself requiring our aid.

Within these limits epistaxis presents varieties according to the cause producing it. Thus we have epistaxis from congestion or over-action; different species of passive epistaxis, as the *scorbutic*, *chloratic*, *&c.*; Epistaxis *supplementary* or substitutive, hemorrhages, which, by their abundance, often occasion serious accidents, and even death. We have examples often of these sad terminations in episiaxis *idiopathic*, as well as symptomatic. Kinds that ought to attract our attention, and to be watched that they do not go beyond certain limits. This hemorrhage has been divided into critical and symptomatic, with or without fever, or into plethoric, febrile, critical, and passive. These distinctions are of practical importance.

All kinds of nasal hemorrhages now are designated as Epistaxis; anciently it was called "*Hæmoragia* Narium, or simple *Hæmorrhagia.*"

Epistaxis is, without doubt, the most frequent of all hemorrhages, but is fortunately rarely so abundant as to impair the general health, or to claim immediate medical aid; such cases do occur however. Few authors have not cited cases of serious import and demanding energetic remedies, and of such a nature as requires us to be acquainted with, and to know the means by which the perils of such cases are to be met.

PREDISPOSING CAUSES—EPISTAXIS IDIOPATHIC.—It is a fact well known, that this is most common in time of youth, yet it is necessary to draw a distinction. If we consider epistaxis in general, without regarding circumstances which give it degrees of gravity very different, no doubt that adolescence and infancy ought not to be the epoch of life when it ought to show its preference. How many individuals do we not find who after having had, during their whole youth, very frequent epistaxis, have seen it disappear, little by little, as they advance to the age of maturity? But if we consider but epistaxis, which by its gravity, compromises the health, nay even the life of its subjects, we are very far from finding, in youth, that proportion of cases, as where it was observed as a slight disease. Of fourteen cases, six were not infants but young men, and eight were at mature age, or advanced in life.

As to Sex.—Facts prove it to have a great influence in the production of this malady. All authors have observed it as much more frequent in males than females; this is true of this disease generally, as well as in grave cases. Of seventeen cases, twelve were males.

It is often Hereditary.—A strong constitution, sanguine temperament, and a plethoric habit are all regarded as predisposing causes. Of nineteen cases, six were accompanied with these indications.

An exciting regimen, abundant alcoholic drinks are often causes; not as frequent, however, as we might suppose.

Season.—Authors do not agree as to this. It is thought, however, that spring and summer, more than other seasons, predispose. Sydenham thought season had no influence, but experience, we think, proves that the warmer seasons have this influence: sedentariness, fleshiness, short neck, amputation of a limb, &c., have, without sufficient proof, been assigned as causes.

Epistaxis Symptomatic.—It is evident that it is to causes predisposing to some maladies, of which this is a symptom; that we must seek for the cause of the symptom. It is consequently unnecessary to extend observations here. In advanced life many cases of this kind are produced. A glance of the eye will show many authors to have noticed this fact, which our own observation had already pronounced.

Cases of epistaxis have been observed in subjects predisposed much to hemorrhages, that it followed losses of blood in numerous ways, or times, or at short intervals. The causes of this hemorrhagic diathesis has not been discovered, and has been named *constitutional* hemorrhage, proving the fact, but without throwing light upon it. These epistaxis then, are they due to a particular state of the blood? to an exaggerated congestion of the mucous membrane? to its too great permeability? These are problems which it is impossible to solve.

OCCASIONAL CAUSES.—If we have taken the pains to find the predisposing causes, we have still to do to discover the occasional causes. I know most authors have given a long list of them, and had we been satisfied with mere assertion, we might have been content; but if proof is wanted, we can find little. Of nineteen cases, which had been well observed, only six could be referred to the occasional causes of the hemorrhage.

There has also been noticed *epidemical* epistaxis. Morgagni mentions one which destroyed a great number of persons of Eutruria and of Romagna, but gives no details by which we can determine whether it was idiopathic or symptomatic of some general disease.

SYMPTOMATIC EPISTAXIS.—The occasional causes of this kind are not observed to differ from those producing the other forms, only in general they are much more powerful. This is all we can say of it, in the present state of the science.

SYMPTOMS.—Is it necessary, in order to study the symptoms, to divide epistaxis into idiopathic and symptomatic? It is necessary to establish this division. But when it acts itself but as a flow of blood which, by its abundance, and the accidents which have followed it, acquires the importance of a particular malady, we may consider the symptoms, independent of their special cause, always taking care to indicate that cause, when it presents itself.

It is unnecessary to display at length, that form of epistaxis which occurs so frequently in infancy and youth, but which is considered by no one as a true malady. It will suffice to say a word of it when we shall have to speak of the effects produced by the suppression of nasal hemorrhage. What use is there of an extended account of the epistaxis of febrile diseases, otherwise than as a part of their history?

INITIATORY SYMPTOMS.—There is no hemorrhage where we have more frequently found the "Molimen Hæmorrhagicum" than epistaxis.

The ancients have described, with some care, these precursory symptoms; but as they established no distinction between the different species of epistaxis, except those of febrile maladies, it follows that amongst these symptoms a certain number appertain to the principal disease of which epistaxis is but a symptom. In a typhoid fever, for example, we cannot say whence supervenes the epistaxis, as that hæmorrhage has had as symptoms, cephalalgia, vertigoes, drainings of the ears, general feebleness, &c., because these symptoms are those which introduce the fever itself.

The incipient symptoms now admitted are the following: itching of the interior of the nares, increased heat, dryness of the mucous, stoppage in the nose, heaviness at its root, frequent sneezing—sometimes a sense as of a strange body in the nasal passage, beating of temporal arteries, congestion of the face, brightness of the eyes, hearing hard, buzzing or whistling of ears, inactivity, heaviness of head, cephalalgia, hardness of pulse, coolness of extremities, &c., &c. We find it said by some authors, that the symptoms are seated in but one side of face; but facts are wanting to prove this.

It is certain in many cases, we establish but one or several of the many symptoms which have been indicated; but when we examine attentively the facts, we soon are convinced that it is a picture most exaggerated, and we see but a relic of that confusion which conducted the ancients to attribute to one malady what belonged to another. How often do we see epistaxis occurring without any of these signs.

It is this which has been recognized by Dr. Kerr and M. Rochoux, who thus expresses himself: "Where we observe truly the grave and numerous accidents, it is especially in acute affections, susceptible of presenting epistaxis, either as a crisis or complication; that the symptoms which announce it, the species of storm which precedes it, and during which it is affected, belong much more, evidently, to the principal disease than to the particular hæmorrhagic effort."

Epistaxis, though slight, may be sufficiently grave to effect seriously the body. Its premonitory symptoms are exceedingly various, nor are they in proportion to the seriousness of the malady often.

In one we will have a strong pulse; in another, brilliant eyes, hard and accelerated pulse; in a third dizziness or stupor; in a fourth we have heaviness and slight pain of head, &c., &c.

It has been divided into Active and Passive or Arterial and Venus.

Active epistaxis have most frequently one or more of the symptoms indicated, they have been regarded as characteristic. There is a distinction to be made by some means, which must naturally affect our treatment.

SYMPTOMS DURING THE HÆMORRHAGE.—The principal one, of course, is the flow of blood from the nasal passages; but we are to look also to the *amount*, the *physical properties* of the blood lost, and the manner in which it escapes. The manner in which the blood flows, has by some authorities, been thought to justify the distinction into Arterial and Venus epistaxis.

One or more of the symptoms enumerated above, having continued for a longer or shorter period, (this being uncertain,) or indeed sometimes without any premonition, the hæmorrhage commences, drop by drop perhaps, sometimes by a sudden gush; it escapes from the anterior nasal openings, or more rarely both these and the posterior passages, or more rarely still, by the latter alone.

The impetuosity with which the blood flows has, by some, been made a test of the active or passive nature of the hæmorrhage. This is not, as a general rule, reliable. The flow may be equal in rapidity in both forms of the disease.

The color, temperature, and coagulability have all been noted by some authors as indications upon this point also. They say when the blood comes out red, warm, and actively coagulable, we may conclude it of the *active form;* the marked diminution ot which, in either of these qualities, indicating the *passive form.* These opinions want the support of sufficient facts, however.

The amount of the sanguinous loss is very variable in mere bleeding of the nose, which rarely attracts the attention of the physician; the loss is of little importance, unless from its frequency, course, or suppression; but in the more grave hæmorrhages the loss of blood from the same parts may be very enormous, from six to as much as twelve pounds being reported as having escaped. Some writers state that excessive nasal hæmorrhages belong to that called *passive* or symptomatic, but it is found that of nineteen cases, six gave evidences of their active nature, such as redness of face, headache, fullness, hardness of pulse, &c.

The precise point from whence the infiltration of blood takes place is not always easily determined, most frequently it is located in the anterior half of the nasal fossæ; sometimes the posterior, and again, extends itself above the whole membrane. Some say it rarely comes from the membrane higher than that which lines the allæ nasi and the convexities of the inferior turbunated bones, so that it may almost always be seen by an attentive examination of these regions. This is not generally true, perhaps. Its issue is usually at the nearest outlet to its seat. This matter is of course affected by position. If the person is standing with the head inclined forward, a hæmorrhage seated even in the posterior naries would run from the facial opening, or if lying on the back, into the pharynx and be thrown from the mouth, and in some cases may descend almost into the stomach, be ejected by an effort like vomiting, and be mistaken for hæmatamesis.

Epistaxis sometimes is suddenly suspended by the formation of clots, in the anterior naries, but is soon renewed by their removal, or it may thus be directed backwards and into the æsophagus, and thus cause bloody vomiting.

When this hæmorrhage attacks a person of good constitution, strong, plethoric, and given to congestions of the head, the first effects are notable, alleviation of the symptoms, and if the flow is not too abundant, the best effects result; but these flows are usually of moderate amount. If it is prolonged, symptoms may supervene which indicate danger, and that show it to be symptomatic itself, and especially where the patient is of feeble constitution, chloratic, scorbutic, &c.

In cases occurring in those weakly constituted persons, exhaustion to the last degree, is observed, there will be pale face for a length of time after, the general integuments will be discolored, the extremities cool, horripilations, cold sweats, position on the back, great effort to move, sometimes prostrate upon the ground in his own blood from a sudden attack, conscious but helpless.

In other cases, the loss of blood, although too small to cause such exhaustion or such grave accidents, are sufficiently so to leave after them, languor, feebleness, palpitation of the heart from slight causes, inappetency, difficult digestion, and general indications of morbid nervous susceptibility. The repetition of this form of hæmorrhage may, of course, produce all these unhappy effects, though the several attacks may not have been so abundant as to induce at the periods of their occurrence, alarm as to such a result.

Some examples are reported of cases attended with petechæ, and ecchymoses on different parts of body; these are very grave and of great violence.

It is also important to remember that these are cases which are not sufficiently bountiful, leaving symptoms which preceded the hæmorrhage unrelieved. They are too soon and too suddenly arrested, and we are required to adopt measures to supply this deficiency.

COURSE, DURATION, AND TERMINATION.—It is known that slight nasal hæmorrhages occur frequently in persons, at intervals of more or less duration, for many years without serious results. Sometimes this may be the case for a lifetime. It more frequently, however, ceases after puberty. Again, it may recur periodically, every month or every season, and so become a necessary drain, whose interruption affects the comfort of the subject, the head or other parts complaining on its stoppage. In other cases it has assumed a quotedian or quarter habit, lasting several hours at each period, and then ceasing. In such cases, their intimate relation to intermittent fever was seen in their arrestation by Sulph. Quinine administered as for the fever.

These hæmorrhages, when so abundant as to cause danger, rarely continue uninterruptedly to flow. The blood escapes with some force for a time, but will cease, regain, as it were, power and again start with new activity. It is in passive bleeding that the flow is most uniform. The duration is a little uncertain, from a few minutes to several hours. Usually fifteen or twenty minutes is the time of an attack, where it is not very grave; if it be, it is often prolonged. Without estimating the irregular interruptions during the flow, we may say, in serious causes, that from five to twenty-four hours will be the duration, but if we consider the time of each flux then, more or less frequent as they may be, three or four hours may be regarded as excessive, and in such the symptoms become alarming.

When the epistaxis terminates happily, we observe the bleeding to diminish gradually, without the use of topical remedial, and the general symptoms to disappear in the same way. Thus the heat will reappear in the extremities, the pulse will rise, the depression subside. When the means used suppress, more or less suddenly, the hæmorrhage, we see the same indications of improvement. Should, however, the blood have been lost in large quantities, the exhaustion will leave its impression, more or less, for some time, and the patient slowly regains his usual state of health.

PATHOLOGY.—Tumefaction and injection of mucous membrane —when chronic, a varicose state of the capillaries has been seen, sometimes thickening of membrane, nasal fossæ and maxillary sinuses have been found filled with clots; sometimes no lesion is observed, and we can scarcely say there is any lesion peculiar to it. In some, at different points of the integuments, ecchymosis, more or less deep, have been found, due to infiltration of the blood into the sub-cuticular cellular tissue; in others, simple petechæ. In one case, sanguinous bloches were observed in all the organs, even in the parieties of the heart. In such extreme cases, no doubt, the blood itself is altered.

